# Can concomitant bladder neck incision and primary valve ablation reduce early re-admission rate and secondary intervention?

**DOI:** 10.1590/S1677-5538.IBJU.2021.0383

**Published:** 2022-01-28

**Authors:** Ahmed Abdelhalim, Abdelwahab Hashem, Ebrahim E. Abouelenein, Ahmed M. Atwa, Mohamed Soltan, Ashraf T. Hafez, Mohamed S. Dawaba, Tamer E. Helmy

**Affiliations:** 1 Mansoura University Urology and Nephrology Center Department of Pediatric Urology Mansoura Egypt Department of Pediatric Urology, Urology and Nephrology Center, Mansoura University, Mansoura, Egypt; 2 National Nephrology and Urology Institute Department of Urology Cairo Egypt Department of Urology, National Nephrology and Urology Institute, Cairo, Egypt; 3 International Medical Center Department of Urology Cairo Egypt Department of Urology, International Medical Center, Cairo, Egypt

**Keywords:** Urinary Bladder Neck Obstruction, Ablation Techniques, Urinary Bladder, Neurogenic

## Abstract

**Objective:**

To assess the effect of bladder neck morphology and its incision (BNI) in patients with posterior urethral valve (PUV) on early reintervention rate.

**Patients and methods:**

Infants undergoing PUV ablation (PVA) before 24 months of age and had at least 18 months of follow-up, were categorized into three groups according to the bladder neck appearance on baseline radiological and endoscopic examination: group 1; normal bladder neck underwent PVA, group 2; high bladder neck underwent PVA plus BNI, group 3; high bladder neck underwent PVA only. Early reintervention was defined as the need for check cystoscopy because of persistent renal function deterioration, worsening hydronephrosis and/or unsatisfactory VCUG improvement during the 1st six months post primary PVA.

**Results:**

Between 2000 and 2017, a total of 114 patients underwent PVA and met the study criteria with a median follow-up of 58 (18-230) months. For group 1, 16 (22.9%) patients needed readmission. Check cystoscopy was free and no further intervention was performed in 5(7.5%) and re-ablation was performed in 11(15.7%) patients. For group 2, 3(14.3%) patients needed reintervention. Re-ablation and re-ablation plus BNI were performed in 1(4.8%) and 2(9.5%), respectively. For group 3, cystoscopy was free in 1(4.3%), re-ablation and re-ablation plus BNI were performed 2(8.7%) and 1(4.3%), respectively. There were no significant differences in the re-admission and re-intervention rates among the three study groups (p=0.65 and p=0.50, respectively).

**Conclusion:**

In morphologically high bladder neck associated PUV, concomitant BNI with PVA doesn’t reduce early re-intervention rate.

## INTRODUCTION

Endoscopic ablation (PVA) remains the gold standard treatment of posterior urethral valves (PUV). However, bladder dysfunction persists in 75-80% of the affected boys even after successful PVA (1). A significant proportion of boys treated for PUV have high riding bladder neck (BN) that could potentially result in secondary bladder neck obstruction, obstructive voiding pattern, gradual detrusor decompensation and eventually myogenic failure, despite early and adequate valve ablation (2).

The diagnosis of BN obstruction in children with PUV is not straightforward. Waterhouse reported that the BN is usually not narrow, but it only has a “pseudo-narrow” appearance; as it lies between the bladder and the dilated proximal urethra (3). Glassberg and Combs believed that the diagnosis of secondary BN obstruction should be based on videourodynamics with elevated voiding pressure, obstructed uroflow pattern and a silent electromyography. Such a diagnosis should not be solely made based on endoscopic examination or radiological findings (4).

While some authors assume that high riding BN is secondary to PUV obstruction, others believe that concomitant primary BN obstruction could exist in some patients. Even more, long-standing PUV obstruction may result in BN remodelling with subsequent BN obstruction. In that instance, PUV ablation alone my not be sufficient to relieve obstruction. Various treatment options have been proposed to target BN obstruction in PUV patients, including alpha blockers (5–7), bladder neck incision (BNI), BN botulinum toxin injection and clean intermittent catheterization (CIC) (4, 8, 9). Glassberg and Combs favored alpha blocker therapy to BNI; as the effects of alpha blocker treatment are reversible with treatment discontinuation (4). However, data on the safety and efficacy of alpha blockers in children with PUV are lacking. BN botulinum toxin injection failed to improve bladder dynamics or enhance hydronephrosis or vesicoureteral reflux (VUR) resolution in a prospective study by Mokhless et al. (9). Finally, CIC is difficult to establish in boys with PUV owing to sensate urethras and bladder neck hypertrophy that often renders catheterizations challenging.

The outcomes of synchronous PVA and BNI are debatable (8, 10). Kajbafzadeh et al. reported 28% reintervention rate with valve re-ablation and/or BNI when patients were treated with PVA only, versus 0% reintervention rate when BNI was concomitantly performed with PVA. Furthermore, concomitant PVA and BNI decreased the need for anticholinergic therapy and CIC, thereby effectively reducing treatment costs and morbidity (8). It has become clear that unplanned re-admissions and secondary procedures following urologic surgery in children negatively affect the quality of medical care and health care expenses (11). In contrast to Kajbafzadeh’s report, Singh et al. reported improved peak flow rates and postvoid residual; but similar compliance, detrusor overactivity, end-filling pressure, maximum Pdet at Qmax and reflux resolution rate in a prospective randomized study comparing PVA alone to concomitant PVA and BNI (12). In this study, we aim to assess the effect of BN status and its incision in PUV patients on the early reintervention rate compared to PVA alone. We believe that bladder neck obstruction in PUV is a secondary phenomenon that would improve after PVA alone. Therefore, adjunctive BNI may not be necessary. Contrary to the conclusion made by Kajbafzadeh et al., we assume that concomitant PVA and BNI does not decrease the need for early reintervention in patients with PUV.

## PATIENTS AND METHODS

After IRB approval (R.21.07.1384), the database of a tertiary centre was retrospectively reviewed for infants (younger than 2 years of age) diagnosed with and treated for PUV between January 2000 and December 2017. Only patients with at least 18 months of follow-up were included. Patients with other anomalies that could potentially affect the lower urinary tract function and those with history of urinary diversion (e.g., vesicostomy or ureterostomy) were excluded. Patients were categorized into three groups according to the BN appearance on baseline radiological and cystoscopic assessment. High BN was judged at the time of cystourethroscopy by an experienced fellowship-trained pediatric urologist by the need for upward deflection of the scope to access the bladder, with or without the presence of bladder neck shelving on the lateral films of voiding cystourethrogram (VCUG). Group-1 included patients without BN elevation who underwent PVA only. Group-2 included patients with high BN who underwent concomitant PVA and BNI and group-3 included patients with high BN who underwent PVA only.

**Figure 1:**
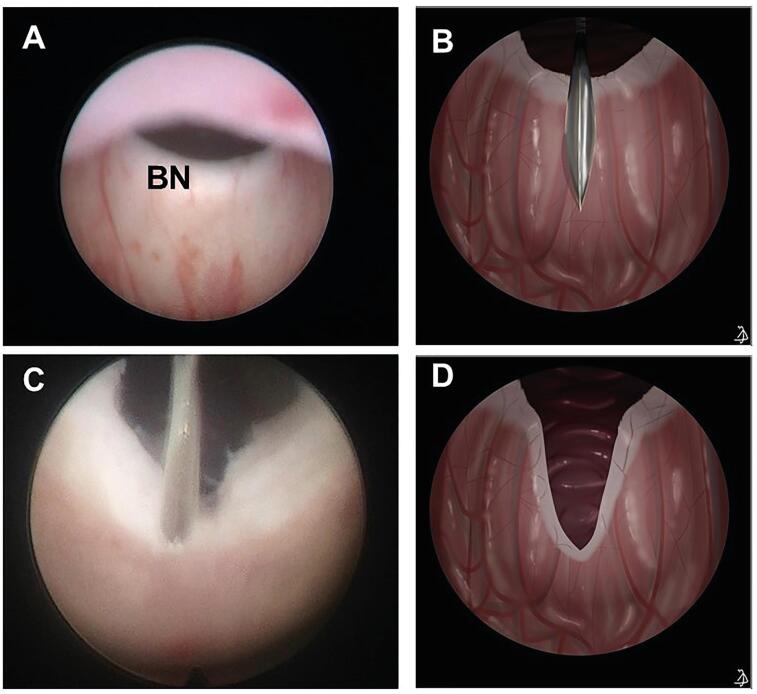
Endoscopic appearance of the hypertrophied bladder neck.

Preoperative evaluation included history, physical examination, serum creatinine, renal bladder ultrasound and VCUG. After initial bladder decompression using a urethral catheter and correction of any existing electrolyte and acid-base imbalance, cystoscopy and PVA were performed in all patients irrespective of serum creatinine level or imaging findings. Surgeries were performed by one of three experienced pediatric urologists with more than 5 years of experience post fellowship. PVA was routinely performed using cold knife urethrotome at 12, 5 and 7 o’clock. If deemed necessary by the operating surgeon, BNI was carried out with an additional single incision also using cold knife at 6 o’clock position stopping proximal to the verumontanum (Figure-1). Adequacy of BNI was confirmed by the ability to visualize the bladder lumen with the tip of the scope at the level of the verumontanum and the expression of an adequate urine stream with Créde manoeuvre.

Patients were followed up at 6 weeks, 3 and 6 months postoperatively and every 6 months thereafter. Follow-up included serum creatinine, ultrasonography on every visit and VCUG at 3 months. The primary study endpoint was the need for reintervention with check cystoscopy within 6 months postoperatively. Reintervention was indicated if there was repeated urine retention, renal function deterioration, worsening hydronephrosis, persistent or worsening dilation of the posterior urethra, worsening or new onset VUR on VCUG. Secondary endpoints were last follow-up serum creatinine and VUR resolution or downgrading.

### Statistical analysis

Data were statistically analysed using the Statistical Package for Social Sciences (SPSS Inc., Chicago, IL, USA, version 20). Qualitative data were described as numbers and percentages. Quantitative data were described as means±SD or medians (ranges), as appropriate. Chi-square test was used to compare categorical data. In the normally and non-normally distributed variables, one-way ANOVA and Kruskal-Wallis test were respectively used for comparisons. P value <0.05 was considered statistically significant.

## RESULTS

Eligible for inclusion were 114 infants who underwent PVA at the study institution at a median age of 7.9 (1–24) months. Median follow-up was 58 (18–230) months. Group-1 (BN not elevated and treated with PVA only) included 70 patients, group-2 (elevated BN treated with concomitant PVA and BNI) included 21 patients, while group-3 (elevated BN treated with PVA only) included 23 patients. Baseline demographics are shown in Table-1. Age at intervention, baseline serum creatinine and baseline VUR were not significantly different between the study groups (p=0.96, 0.95 and 0.42; respectively).

**Table 1:** Baseline patient demographics.

Patient characteristics	Group 1 (BN not elevated and treated with PVA only), N=70	Group 2 (elevated BN treated with concomitant PVA and BNI), N=21	Group 3 (elevated BN treated with PVA only), N=23	P-value
Median age at PVA (range), months	8 (1-24)	7 (1-22)	5.5 (1-24)	0.96
Median follow-up duration (range), months	54 (17 - 230)	44 (18- 136)	49 (22 - 157)	0.14
Median baseline serum creatinine (range), mg/dL	0.5 (0.1-2.2)	0.4 (0.2-1.7)	0.6 (0.2-1.2)	0.95
**Baseline VUR, N (%)**				0.42
No VUR	42 (60)	11 (52.4)	9 (39.1)	
Non-dilating VUR (grade I-II)	2 (2.9)	0	1 (4.3)	
Dilating VUR (grade III- IV)	26 (37.1)	10 (47.6)	13 (56.5)	

**BNI** = bladder neck incision; **PVA** = posterior urethral valve ablation; **VUR** = vesicoureteral reflux

At last follow-up, median serum creatinine was 0.6 (0.3-5.2), 0.5 (0.4-1.4) and 0.5 (0.4-2.4) mg/dL for groups 1, 2 and 3; respectively (p=0.48). Overall, a total of 23 (20.2%) patients required check cystoscopy for repeated urine retention, renal functional deterioration, worsening hydronephrosis or inadequate improvement of VCUG findings. In group-1, 16 (22.9%) patients needed reintervention. Check cystoscopy was unremarkable and no further intervention was required in five (7.5%) cases. Ablation of PUV remnants was performed in 11 (15.7%) patients. In group-2, reintervention was required in three (14.3%) patients: ablation of valve remnants was performed in one patient (4.8%) and BNI was combined with ablation of PUV remnants in two (9.5%) other patients. Reintervention was required in 4 (17.4%) group 3 patients. Of those, check cystoscopy was unremarkable in one patient (4.3%), two patients underwent ablation of PUV remnants (8.7%) and one underwent both BNI and ablation of PUV remnants (4.3%). The reintervention rates and the type of reintervention were not significantly different among the three study groups (p=0.65 and 0.15; respectively). Likewise, follow-up serum creatinine and VUR outcomes were not different among the three study groups. Study results are summarized in Table-2.

**Table 2:** Study outcomes.

Study outcome		Group 1 (BN not elevated and treated with PVA only), N=70	Group 2 (elevated BN treated with concomitant PVA and BNI), N=21	Group 3 (elevated BN treated with PVA only), N=23	P-value
**Number of patients requiring re-intervention within 6 months postoperatively (%)**					0.65
	No	54 (77)	18 (85.7)	19 (82.6)	
	Yes	16 (22.9)	3 (14.3)	4 (17.4)	
**Type of reintervention**					0.15
	No reintervention	54 (77)	18 (85.7)	19 (82.6)	
	Check cystoscopy only	5 (7.5)	0	1 (4.3)	
	Ablation of PUV remnants	11 (15.7)	1 (4.8)	2 (8.7)	
	Ablation of PUV remnants + BNI	0	2 (9.5)	1 (4.3)	
Median last follow-up serum creatinine (range), mg/dL		0.6 (0.3-5.2)	0.5 (0.4-1.4)	0.5 (0.4-2.4)	0.48
**Postoperative VUR, N (%)**					
	No VUR	46 (65.7)	12 (57.1)	11 (47.8)	
	Non-dilating VUR (grade I-II)	3 (4.3)	1 (4.8)	2 (8.7)	0.62
	Dilating VUR (grade III- IV)	21 (30)	8 (38.1)	10 (43.5)	
**VUR outcome (%)**					
	Improved	12 (17.1)	4 (19)	5 (21.7)	0.72
	Static	55 (78.6)	17 (81)	18 (78.3)	
	Worsened	3 (4.3)	0	0	

**BNI** = bladder neck incision; **PUV** = posterior urethral valve; **PVA** = posterior urethral valve ablation; **VUR** = vesicoureteral reflux

## DISCUSSION

PUV is the commonest cause of lower urinary tract obstruction in male children (13). Even after successful PVA, the majority of patients will suffer bladder dysfunction. Following PVA, bladder function greatly varies from essentially normal voiding to severe bladder dysfunction with prolonged and weakened urinary stream. Obstructive voiding pattern can be the result of residual valve tissue, urethral stricture particularly if cautery was used for PVA or secondary BN obstruction (14). Radiological and endoscopic examination usually suffice when evaluating valve remnants and urethral stricture. The diagnosis of secondary bladder neck obstruction is, however, more challenging, often requiring videourodynamic assessment (4).

Management of PUV sequelae is dictated by the pattern of bladder dysfunction (15–17). A variety of therapeutic options have been suggested to treat secondary BN obstruction in PUV patients including alpha-blockers, botulinum toxin injection into the BN or BNI (4, 8, 9). For fear of the long-term consequences of BNI on ejaculation and continence, some pediatric urologists favored the use of alpha blockers to relax the BN and treat secondary BN obstruction. In patients with urodynamic diagnosis of secondary BN obstruction, Combs reported decreased mean maximum voiding detrusor pressure (Pdet) from 107.3 to 41.2cm H2O and increased Qmax from 12.5 to 24.7mL/sec following alpha blocker treatment (7). Mokhless et al. injected botulinum toxin into the BN of 10 patients with BN dysfunction following PVA. The authors of that study found no effect of BN botulinum toxin injection on urodynamics, hydronephrosis or VUR resolution 6 months after the procedure (9).

In the 1950s, BNI was commonly performed during PVA to improve voiding. This practice was later abandoned for fear of incontinence and retrograde ejaculation (3). A number of contemporary studies have examined the effect of concomitant BNI and PVA with conflicting results. Kajbafzadeh et al. described, modified BNI at 6 o’clock position through the BN just proximal to the verumontanum cautiously leaving the adventitia untouched to preserve antegrade ejaculation. In this prospective study, 22 patients underwent concomitant PVA and BNI and 24 matched patients underwent PVA only. At baseline, all patients in both groups had hypercontractile bladders and comparable voiding detrusor pressures. After a mean follow-up of 4.5 years, patients who had concomitant PVA and BNI had a mean maximal voiding Pdet of 53±15cm H2O without any bladder hypercontractility or detrusor overactivity. In the PVA group, the mean maximal voiding Pdet was 87±45cm H2O (p <0.01). Nine (37.5%) patients of that group had bladder hypercontractility and six (25%) had detrusor overactivity (8). In another study by Sarin et al., bladder dysfunction and detrusor overactivity rates were similar whether BNI was simultaneously performed with PVA or not. Hypocompliant high-pressure bladder was the predominant cystometric finding in both groups. Although limited by the small number of subjects and short follow-up, this study failed to demonstrate any additional benefits of simultaneous BNI (10). Singh et al. reported improved peak flow rate and PVR, but similar compliance, DO, end-filling pressure, maximum Pdet at Qmax and reflux resolution rate in a prospective randomized study comparing PVA alone to concomitant PVA and BNI (12).

Few studies have examined the effects of BNI performed during childhood on retrograde ejaculation and urinary continence in adulthood. Taskinen et al. reported ejaculation failure in two of 19 adults who had concomitant PVA and BNI during childhood. However, dry ejaculates were also reported in one of 15 patients treated with PVA only indicating that BNI during childhood does not result in retrograde ejaculation (18). Hennus and co-workers reported on the long-term effects on ejaculation and urinary continence in 40 participants who had unilateral superficial BNI at a mean age of 4.5 years. All men had antegrade ejaculation, 10.8% reported possibly reduced ejaculatory volume and 5.8% had moderate urinary incontinence (19). Likewise, Keihani found no effect of BNI on continence, antegrade ejaculation or semen quality in 18 adult patients who had PVA and BNI during childhood (20). These studies provide reassurance that BNI can be safely performed without deleterious effects on continence or ejaculation.

In Kajbafzadeh’s study, PVA alone was associated with a 28% reintervention rate versus 0% for patients who underwent concomitant PVA and BNI (8). Without a doubt, reduced readmission risk substantially contributes to improved quality of care and reduced treatment-related costs (11). Furthermore, repeated exposure to general anesthesia during infancy and early childhood has been linked to lower motor and social linguistic skills and poorer school performance (21, 22). Even with questionable benefits to bladder dynamics and upper tract outcomes, reduced risk of readmission and repeated interventions would certainly favor concomitant BNI as long as it does not result in adverse long-term consequences. In contrast to the results of Kajbafzadeh’s study, we found that patients treated with concomitant BNI had similar readmission and reintervention rates compared to those treated with PVA only. The re-admission rates were 22.9%, 14.3%, and 17.4% for patients with normal BN, high BN treated with concomitant PVA and BNI and high BN treated with PVA without BNI; respectively. Further, we found no significance difference in re-intervention rates, serum creatinine or VUR outcome among the three study groups. For patients with high BN who underwent concomitant PVA and BNI, re-ablation of valve remnants and re-ablation plus BNI were performed in one (4.8%) and two (9.5%) cases; respectively. For those patients with high BN who had only PVA without BNI, re-ablation of valve remnants was performed in two (8.7%) patients and re-ablation plus BNI were performed in one (4.3%) patient (p=0.15).

Our study has several limitations. In addition to its retrospective design, the decision to perform concomitant BNI was not based on a study plan or an institutional protocol, but rather on surgeon’s evaluation. In addition, the diagnosis of bladder neck obstruction relied on endoscopic and radiologic findings and not on urodynamic evaluation. The lack of urodynamic evaluation could be regarded as one of this study limitations. Although urodynamics is the best tool we currently have in hand to assess lower urinary tract functions, it is not without limitations. Urodynamics before PVA would not reliably distinguish whether the obstructive voiding pattern is the result of valves or BN obstruction until valve ablation is carried out. Further, patients with PUV have sensate urethras and hypertrophied BN making catheterization sometimes challenging and resulting in significant patient discomfort and possibly affecting the quality of urodynamic results. Even the smallest urodynamic catheter available (usually 6 French) could be obstructive to the infantile urethra, leading to inaccurate interpretation of voiding cystometry. Likewise, the lowest possible bladder filling rate provided by the current urodynamic machines is probably supra physiologic for infants and young children. Moreover, most PUV patients have VUR, usually of high grade, resulting in inaccurate estimation of the bladder capacity and compliance. Parental separation, catheterization and bladder filling all contribute to patient irritability and discomfort resulting in multiple motion artefacts and frequent urine leaks with subsequent difficulty in interpreting the urodynamic tracing. After all, interpretation of urodynamic results is also subject to inter-rate and intra-rate variability even among experts in this field (23). Despite these limitations of urodynamic studies, they remain fundamental in assessment of bladder dysfunction. Although the follow-up duration is relatively short (median of 58 months with a minimum of 18 months), this duration is quite sufficient to judge the short-term outcomes such as the need for early reintervention.

## CONCLUSION

At least in our results, concomitant bladder neck incision with valve ablation in infants with PUV is not associated with reduced early hospital re-admission or re-intervention rates compared to PVA alone. Our results require validation in a larger number of subjects examined in a prospective fashion.
